# Supplementary material for: New Functions of Intracellular LOXL2: Modulation of RNA-Binding Proteins
Source: Molecules. 2023 May 30;28(11):4433. doi: 10.3390/molecules28114433 (PMC10254187; doi:10.3390/molecules28114433)
Supplement: Supplementary file 1 [file molecules-28-04433-s001.zip › Eraso et al., supplementary materials.pdf]

Supplementary materials

Supplementary Datasets.

Dataset S1. Data of LC-MS/MS of immunoprecipitation 1 and 2.

Dataset S2. RNA sequence analysis of the gene expression profile of shLOXL2 silenced cells versus shSCR control cells.

Dataset S3. List of bibliographic references with experimental evidences of involvement in EMT of the EMT-related gene signature.

Supplementary figure legends.

Figure S1. Silencing of LOXL2 in Hs578T and MDA-MB-231 cell lines. LOXL2 silencing using shRNAs (shLOXL2) and a scramble control (shSCR) sequence was confirmed by qPCR (left panel) and WB (right panel).

Figure S2. Distribution of RBP targets among EMT-related DEGs. Red or grey squares denoted the presence or absence of RBPs binding sites respectively

Figure S3. Distribution of RBP targets among no EMT-related DEGs. Red or grey squares denoted the presence or absence of RBPs binding sites respectively.

Supplementary table.

Table S1. Number of genes targeted by the selected RBPs in EMT- and no EMT-related signatures.

Table S2. List of primers used for qPCR analyses.

Table S1

Table S1. Distribution of genes targeted by the indicated RBP in EMT-related and no EMT-related signatures. The p-value was obtained by analysing the enrichment of RBPs hits between both categories using the Fisher's exact test.

RBP	EMT-related	no EMT-related	p-value
**CSTF2T**	16	46	0.1775
**DDX3X**	0	0	na
**DDX54**	24	71	0.1213
**DGCR8**	18	72	0.8854
**DHX9**	47	176	0.4586
**ELAVL1**	42	77	4.1E-06
**FMR1**	75	258	0.0327
**FUS**	12	40	0.4659
**HNRNPA1**	85	180	0.137
**HNRNPC**	30	99	0.212
**HNRNPK**	8	26	0.5064
**IGF2BP1**	21	53	0.0419
**IGF2BP2**	31	104	0.2673
**IGF2BP3**	18	52	0.1514
**LIN28B**	13	35	0.1817
**METTL3**	0	0	na
**MOV10**	15	37	1.000
**RBFOX**	39	134	0.2143
**SRSF1**	32	95	0.0175
**TAF15**	68	224	0.0248
**TARDBP**	30	127	1.000
**U2AF2**	59	192	0.0381
**WTAP**	0	0	na
**YTHDC1**	3	8	0.4528

Table S2

Table S2: list of primers used for qPCR analysis.

Gene	Forward primer (5'→3')	Reverse primer (5'→3')
AKR1C2	CAGAGGTTCCTAAAAGTAAAGC	TGTGCAGAATCAATATGGTG
CCL2	AGACTAACCCAGAAACATCC	ATTGATTGCATCCTGGCTG
CLDN1	TTGGCATGAAGTGTATGAAG	ACCTGCAAGAAGAAATATCG
CXCL3	CCTCAAGAACATCCAAAGTG	CCCCTTGTTCAGTATCTTTTC
DUSP6	TTCTACCTGGAAGATGAAGC	CAATGTCATGGCATCGTTC
ENPP2	ATAAAACAGTACGTGGAAGG	CTACCCATTTTGATTCGTCC
GAPDH	CTTCACCACCATGGAGGAGGC	GGCATGGACTGTGGTCATGAG
HEY2	GGATTATAGAGAAAGGCGTC	GTTTTTCAAAGCAGTTGGC
ID1	ACTAAGTCACCAGAGACTTTAG	AAATCTGAGAAGCACCAAAC
SLC2A3	GGATAACTATAATGGGATGAGC	CCACAATAAACCAGGGAATG
SOD2	ATCATACCCTAATGATCCCAG	AGGACCTTATAGGGTTTTCAG
TULP3	AGGTCCTAGGAAAATGTCTG	AAGTTGAGGACATAGGACTG
