# Supplementary figures and images for: New Functions of Intracellular LOXL2: Modulation of RNA-Binding Proteins
Source: Molecules. 2023 May 30;28(11):4433. doi: 10.3390/molecules28114433 (PMC10254187; doi:10.3390/molecules28114433)

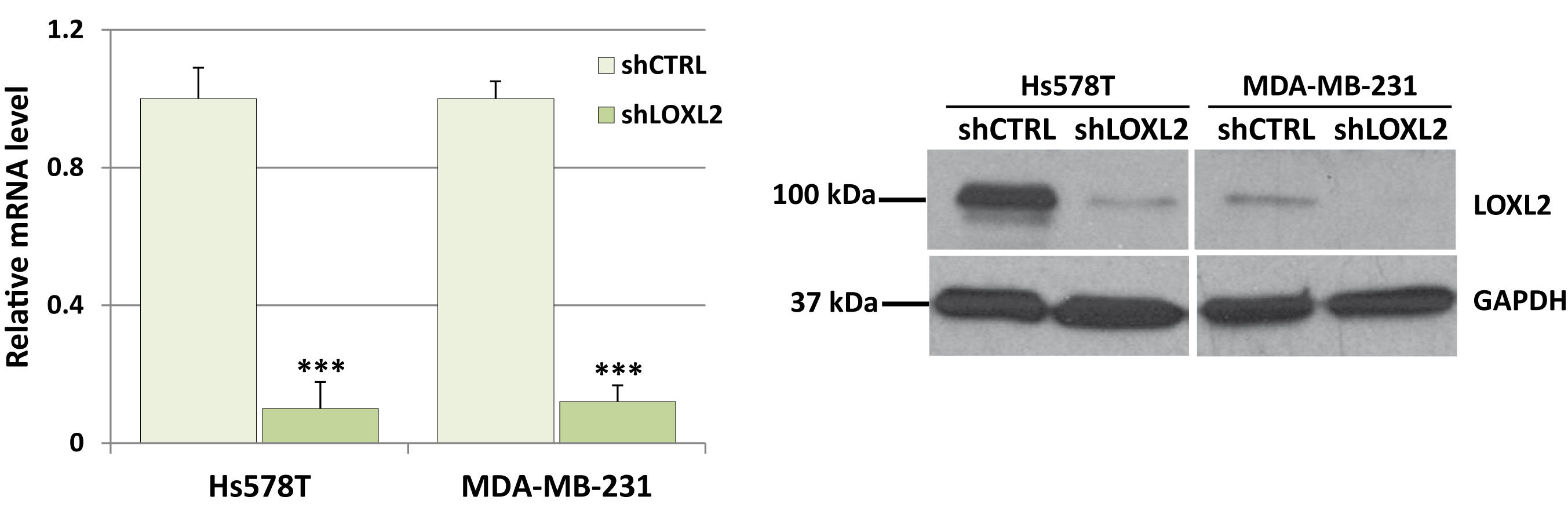

Supplement: Supplementary file 1 [file molecules-28-04433-s001.zip › Figure S1.tif]

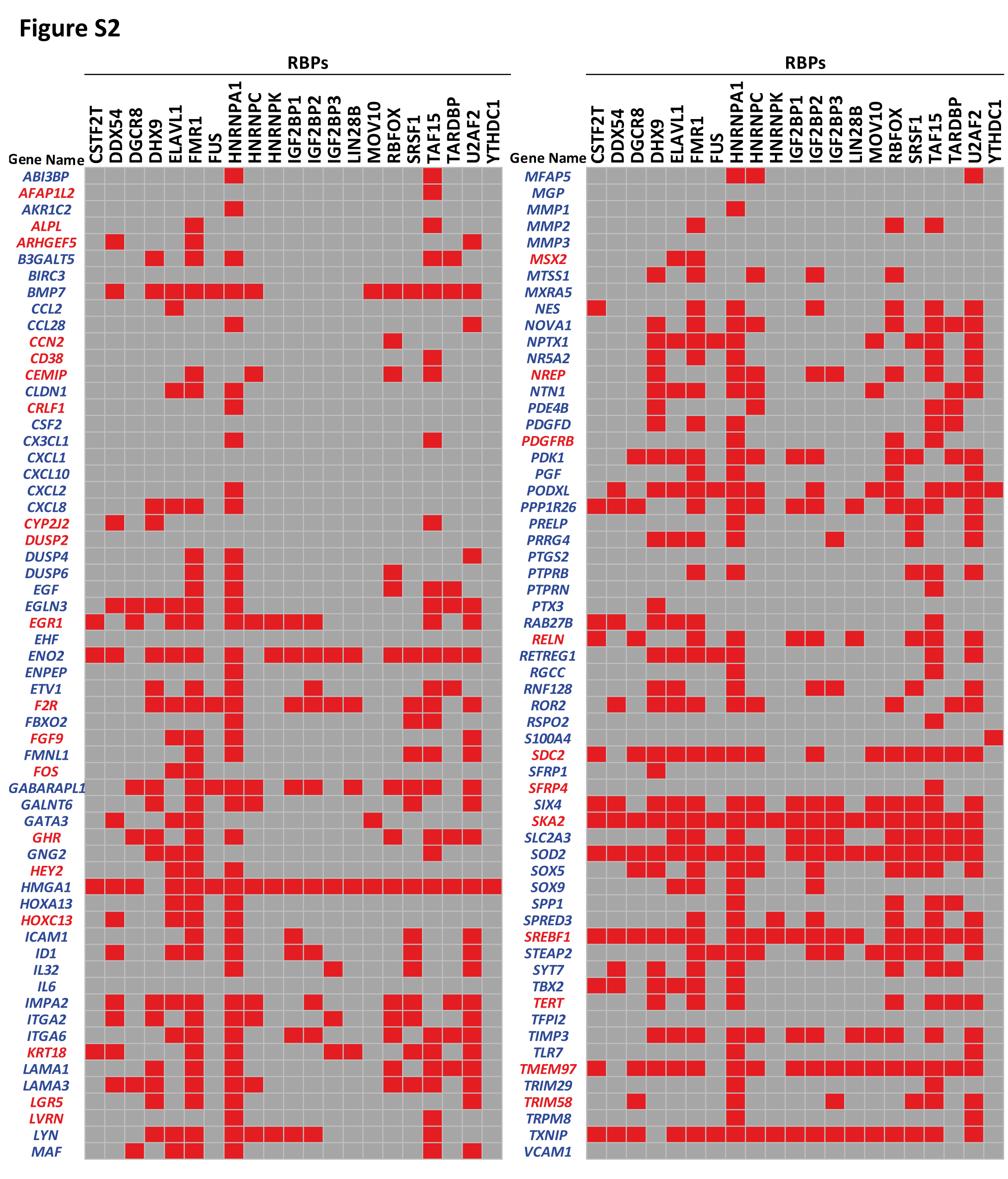

Supplement: Supplementary file 1 [file molecules-28-04433-s001.zip › Figure S2.tif]

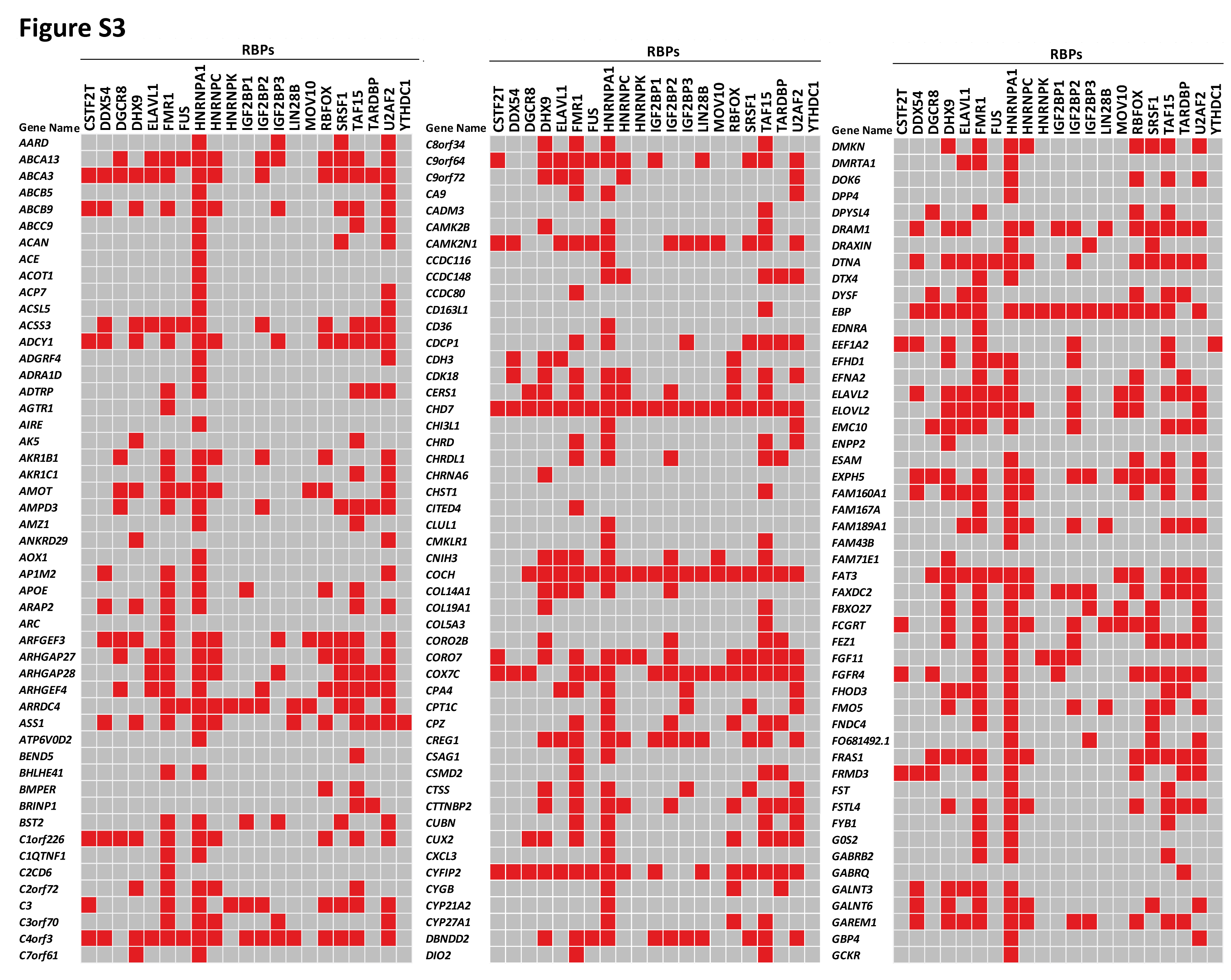

Supplement: Supplementary file 1 [file molecules-28-04433-s001.zip › Figure S3_1.tif]

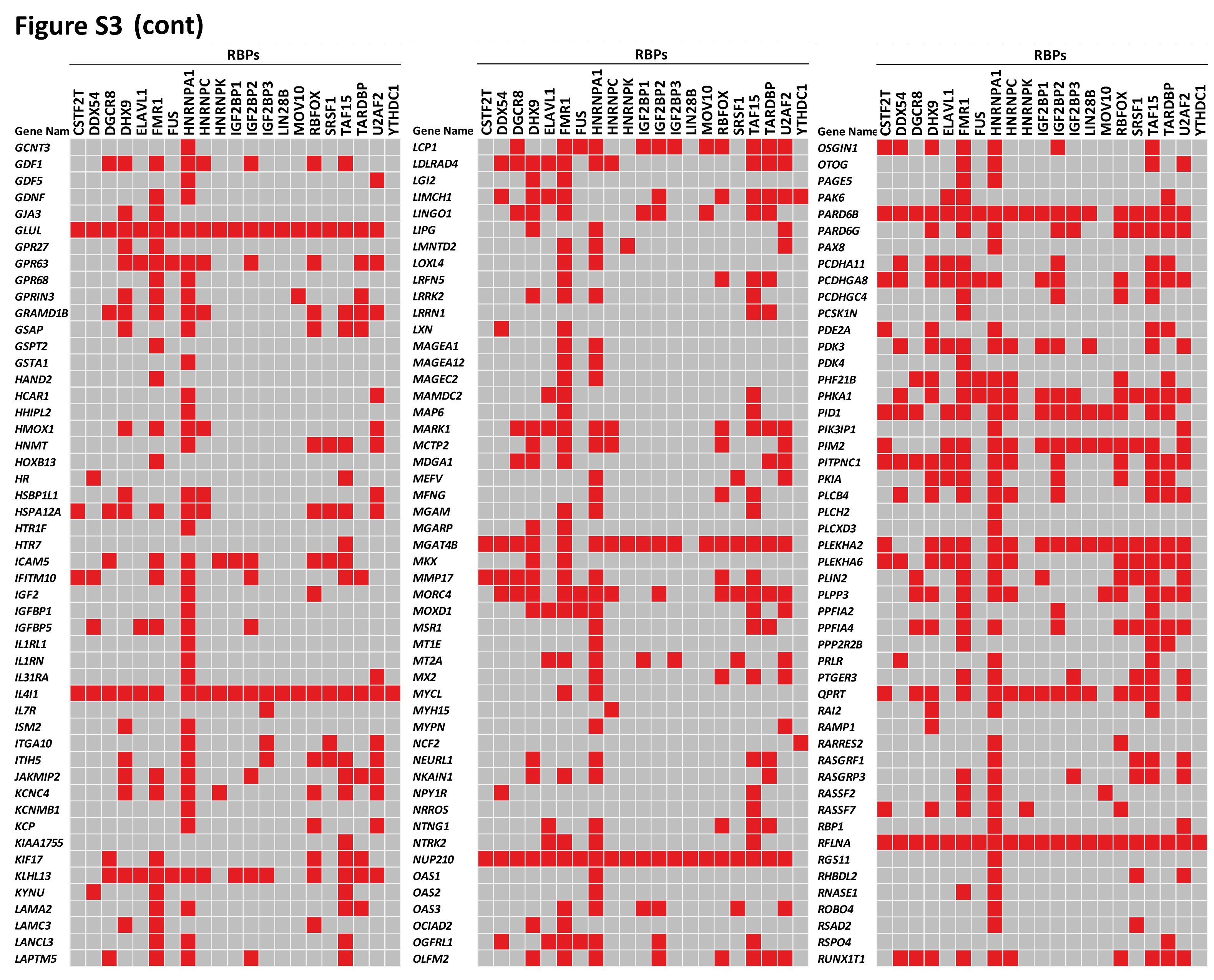

Supplement: Supplementary file 1 [file molecules-28-04433-s001.zip › Figure S3_2.tif]

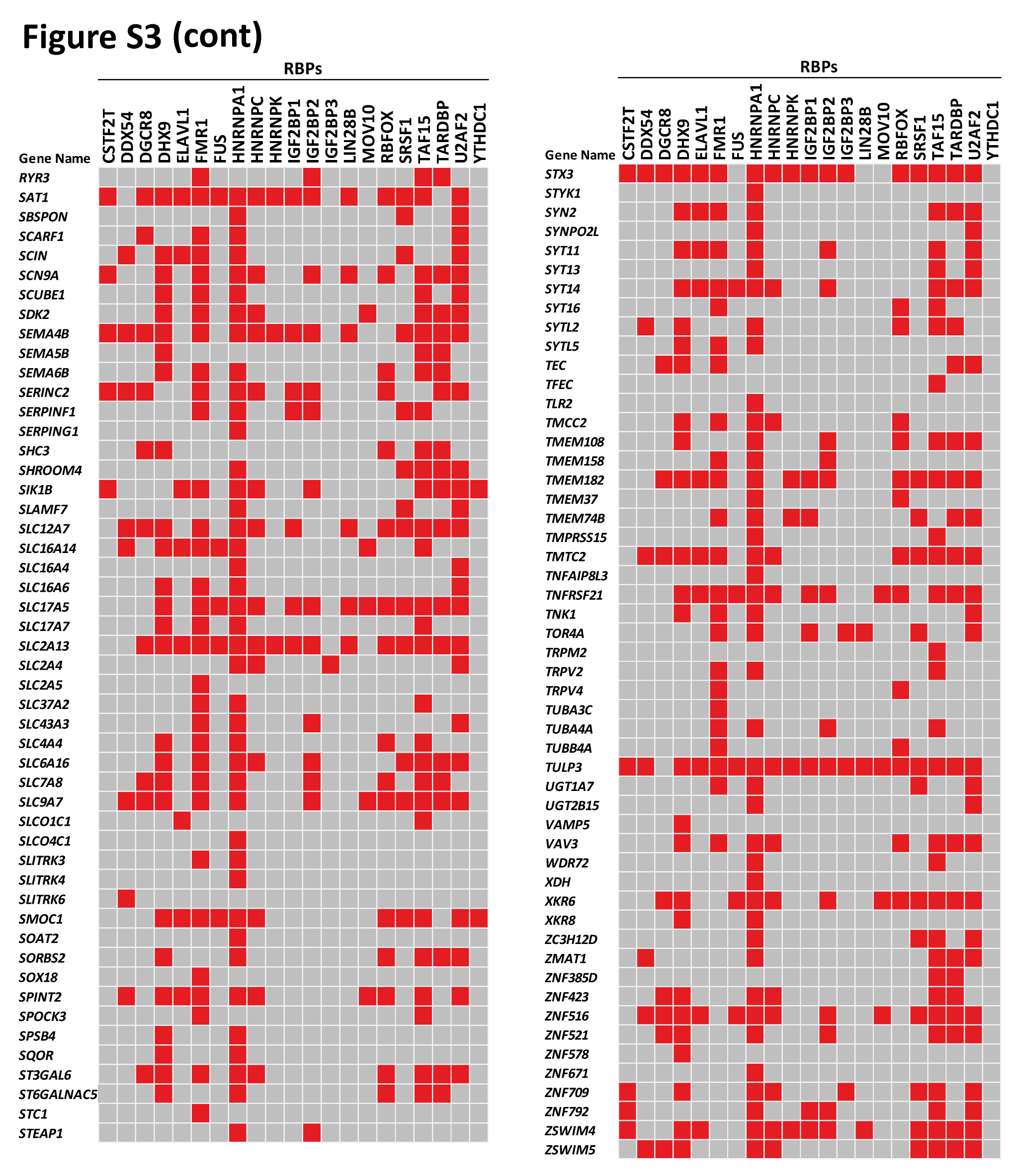

Supplement: Supplementary file 1 [file molecules-28-04433-s001.zip › Figure S3_3.tif]

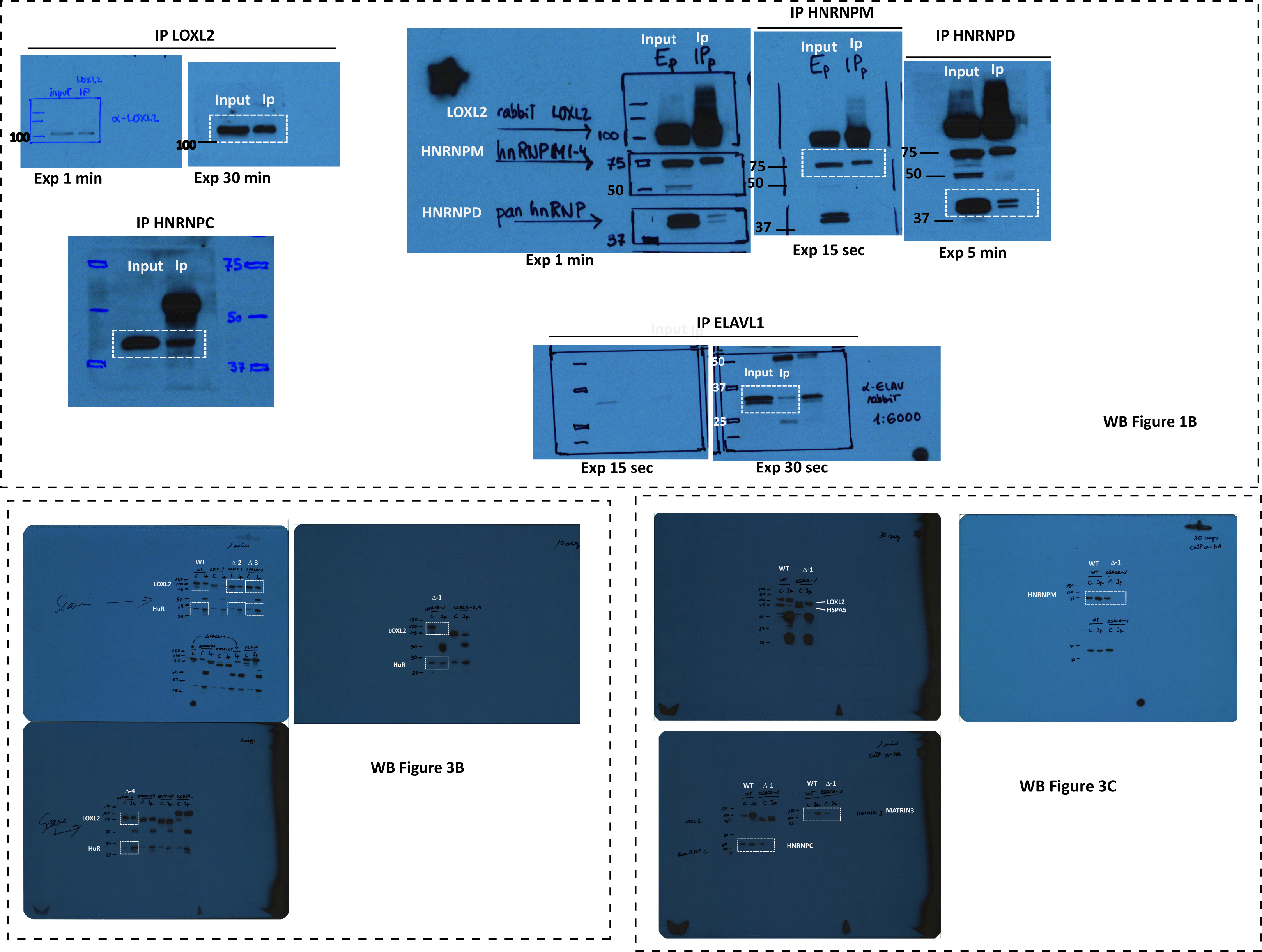

Supplement: Supplementary file 1 [file molecules-28-04433-s001.zip › Supplementary original figures.tif]
